# Long-Term Connectome Analysis Reveals Reshaping of Visual, Spatial Networks in a Model With Vascular Dementia Features

**DOI:** 10.1161/STROKEAHA.121.036997

**Published:** 2022-02-02

**Authors:** Gerard R. Hall, Philipp Boehm-Sturm, Ulrich Dirnagl, Carsten Finke, Marco Foddis, Christoph Harms, Stefan Paul Koch, Joseph Kuchling, Christopher R. Madan, Susanne Mueller, Celeste Sassi, Stamatios N. Sotiropoulos, Rebecca C. Trueman, Marcus D. Wallis, Ferah Yildirim, Tracy D. Farr

**Affiliations:** 1 School of Life Sciences, University of Nottingham, United Kingdom (G.R.H., R.C.T., M.D.W., T.D.F.).; 2 Department of Experimental Neurology, Center for Stroke Research Berlin, Charité-Universitätsmedizin Berlin (P.B.-S., U.D., M.F., C.H., S.P.K., S.M., C.S., T.D.F.), corporate member of Freie Universität Berlin, Humboldt-Universität zu Berlin and Berlin Institute of Health, Germany.; 3 Experimental and Clinical Research Center, Max Delbrueck Center for Molecular Medicine and Charité-Universitätsmedizin Berlin (J.K.), corporate member of Freie Universität Berlin, Humboldt-Universität zu Berlin and Berlin Institute of Health, Germany.; 4 NeuroCure Cluster of Excellence and Charité Core Facility 7T Experimental MRIs (P.B.-S., U.D., M.F., C.H., S.P.K., S.M., C.S., F.Y., T.D.F.), Charité-Universitätsmedizin Berlin, Germany.; 5 Department of Neurology (C.F., J.K.), Charité-Universitätsmedizin Berlin, Germany.; 6 NeuroCure Cluster of Excellence and Department of Psychiatry and Psychotherapy (F.Y.), Charité-Universitätsmedizin Berlin, Germany.; 7 German Center for Neurodegenerative Diseases, Berlin Site, Germany (U.D.).; 8 Berlin School of Mind and Brain, Humboldt Universität zu Berlin, Germany (C.F.).; 9 School of Psychology, University of Nottingham, United Kingdom (C.R.M.).; 10 Sir Peter Mansfield Imaging Centre, School of Medicine, University of Nottingham, United Kingdom (S.N.S.).; 11 Centre for Functional MRI of the Brain, University of Oxford, United Kingdom (S.N.S.).

**Keywords:** connectome, dementia, mice, neuroimaging, white matter

## Abstract

Connectome analysis of neuroimaging data is a rapidly expanding field that offers the potential to diagnose, characterize, and predict neurological disease. Animal models provide insight into biological mechanisms that underpin disease, but connectivity approaches are currently lagging in the rodent.

**Methods:**

We present a pipeline adapted for structural and functional connectivity analysis of the mouse brain, and we tested it in a mouse model of vascular dementia.

**Results:**

We observed lacunar infarctions, microbleeds, and progressive white matter change across 6 months. For the first time, we report that default mode network activity is disrupted in the mouse model. We also identified specific functional circuitry that was vulnerable to vascular stress, including perturbations in a sensorimotor, visual resting state network that were accompanied by deficits in visual and spatial memory tasks.

**Conclusions:**

These findings advance our understanding of the mouse connectome and provide insight into how it can be altered by vascular insufficiency.

Neuroimaging is an important tool to noninvasively characterize the brain, and examination of structural and functional connectomes is rapidly evolving. Connectomics aims to improve our understanding of healthy brain architecture and offers the potential to improve diagnosis, patient stratification, and outcome prediction in disease. Structural connectivity analysis from diffusion magnetic resonance imaging (MRI) may be a useful biomarker for small vessel disease due to the prevalence of white matter change. Reduced global efficiency (a network metric that quantifies the brain’s ability to integrate and process information) has been reported to be a predictor of cognitive decline in patients with radiological features of small vessel disease.^1,2^ This structural network disruption may be accompanied by functional connectivity alterations that can be measured using resting state functional MRI. The default mode network (DMN) refers to a collection of structures with highly correlated activity patterns during internally directed tasks; it is a fundamental brain network that is disrupted in a variety of neurological diseases, including those with vascular origins.^3^ Decreases in functional connectivity in individuals with small vessel disease have been reported between frontoparietal^4^ and frontotemporal regions.^5,6^

Animal models represent a reproducible method to recapitulate features of human disease; they can bridge the gap between macroscale imaging–based connectivity analysis and the microscale processes that underpin it. There is greater translational impact if models replicate imaging biomarkers that predict outcome. However, the size of the rodent brain poses neuroimaging challenges, and connectivity approaches are lagging compared with the progress made in humans. Recent work has linked mouse functional connectivity patterns to distinct axonal projections,^7,8^ and some groups have reported gene-specific connectivity alterations.^9,10^ There are also reports that reduced cortical functional connectivity precedes amyloid plaque deposition in a mouse model of Alzheimer disease^11^ and is correlated with tau aggregation.^12^ The purpose of the present study was to use a novel, hypothesis-free approach to examine functional and structural connectivity change in a mouse model of vascular dementia that is characterized by white matter damage and spatial memory impairments. We hypothesized that known declines in scalar measures of white matter integrity would correlate with a decline in structural connectivity that mimics the clinical scenario, and this would be accompanied by decreases in functional connectivity. Interestingly, despite progressive white matter change, overall structural connectivity remained intact. However, we show long-term functional connectivity alterations in the rodent DMN, as well as cortical hubs associated with the visual and spatial memory system; this was accompanied by behavioral deficits in visual spatial tasks.

## Methods

The data that support the findings of this study are available from the corresponding author upon request. Detailed methods are included in the Supplemental Material.

### Experimental Design

Experiments were performed according to the internal Animal Welfare Committee and the Landesamt für Gesundheit und Soziales (G0068/12) and conformed to the ARRIVE guidelines (Animal Research: Reporting of In Vivo Experiments). Male C57/BL6J mice (8 weeks, Charles River, Germany) were housed under a 12-hour light/dark cycle (temperature, 22±2 °C; humidity, 55±10%) with ad libitum access to food and water. Animals were anaesthetized with isoflurane (70:30 nitrous oxide:oxygen) and body temperature maintained at 37±0.2 °C during all procedures. The surgical procedure involved randomization to groups and wrapping a nonmagnetic microcoil (160 or 500 μm, hypoperfused [n=11] or sham [n=10], respectively; Shannon MicroCoil, Ireland) around a carotid artery. The procedure was repeated for the other carotid artery the following day. Pain relief was provided in the drinking water (6 mg/mL paracetamol) 3 days before surgery and for a week following. All experiments and data analysis were performed blind. There was high mortality in the hypoperfused group: 2 within 48 hours and 3 at 9, 28, and 32 days post-surgery. MRI was conducted at 24 hours (T_2_, cerebral blood flow [CBF]), and 3 (T_2_, CBF, diffusion MRI, magnetic resonance angiography, spectroscopy) and 6 months post-surgery (T_2_, CBF, diffusion MRI, magnetic resonance angiography, spectroscopy, resting state functional MRI). Novel object recognition and the Morris water maze cued and place tasks were performed at 6 months.

### Statistical Analysis

Data are expressed as mean±SD. The Kolmogorov-Smirnov test established distribution (SPSS, IBM, United Kingdom). CBF, volumetry, water maze, and novel object recognition data were compared using mixed 2-way repeated measures ANOVAs. Graph theory metrics were compared with *t* tests or Mann-Whitney *U* tests for resting state functional MRI and mixed 2-way repeated measures ANOVAs for diffusion MRI. False discovery rate significance thresholds were calculated for all ranked *P*-values to correct for multiple comparisons (spectroscopy and local graph theory parameters).

## Results

### Mice With Vascular Cognitive Impairment Display Radiological Features of Small Vessel Disease

**Figure 1.**
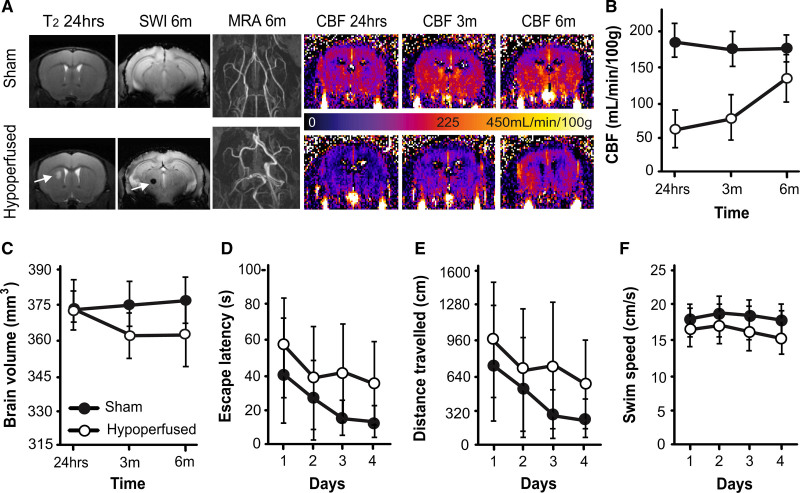
**Hypoperfusion causes lacunar infarction, microbleeds, and brain atrophy. A**, Representative T_2_ and susceptibility weighted image (SWI) at 24 h and 6 mo (arrows indicate lacunar infarction and microbleed), alongside magnetic resonance angiography (MRA) images of the circle of Willis at 6 mo, and cerebral blood flow (CBF) maps at 24 h and 3 and 6 mo (scale bar corresponds to CBF). **B**, CBF (mean±SD) is lower in the hypoperfused group (n=6), and these animals exhibited brain atrophy (**C**). In a water maze cued task at 6 mo, hypoperfused mice exhibited longer escape latencies (**D**), swam greater distances (**E**), and were slower (**F**) than sham mice (n=10).

We observed important radiological features of small vessel disease in hypoperfused mice. Lacunar infarctions were present in 4 of 11 hypoperfused mice at 24 hours, generally in the striatum and occasionally in the white matter, and 1 of 4 did not survive. There was no evidence of T_2_ hyperintensities remaining at 3 or 6 months. Two additional animals exhibited thalamic microbleeds only at 6 months (Figure 1A; Figure S1A). CBF remained stable across 6 months in sham animals but was significantly decreased (by ≈75%) in hypoperfused mice at 24 hours (Figure 1A and 1B). CBF improved between 3 and 6 months in hypoperfused mice but did not reach sham levels (main effect of time: F(2,26)5.2, *P*=0.013; group: F(1,13)133.9, *P*=0.0001; and time×group interaction: F(2,26)7.9, *P*=0.002). We observed the circle of Willis remodeling (increased tortuosity and dilation) in the hypoperfused mice via magnetic resonance angiography (Figure 1A; Figure S1A). The hypoperfused group exhibited significant brain atrophy at 3 and 6 months (time×group interaction: F(2,26)3.6, *P*=0.04; Figure 1C); this was also observed in the hippocampus (Supplemental Results) without changes in structural complexity (Figure S1B and S1C).

### Behavioral Deficits in Mice With Vascular Cognitive Impairment Suggest Visual, Spatial Disturbance

A cued task with a visible platform was performed in the water maze at 6 months. While all mice escaped quicker, and traveled less distance, faster over the 4 test days (main effects of time: F(3,42)13.4, *P*=0.0001; F(3,42)11.7, *P*=0.0001; F(3,42)3.4, *P*=0.027, respectively), the hypoperfused mice exhibited impairments (Figure 1D through 1F). They took significantly longer to escape, traveled greater distances, and swam slower than shams (main effects of group: F(1,14)37.6, *P*=0.0001; F(1,14)33.8, *P*=0.0001; F(1,14)7.8, *P*=0.015, respectively; Figure 1D through 1F). Similar results were observed in a place task in which the platform was hidden in a fixed location (Figure S2A through S2C). Furthermore, hypoperfused mice made significantly fewer returns to the target quadrant during the probe trial in which the platform was removed (Figure S2E).

### Characterization of the Functional Mouse Connectome Depicts Resting State Networks

**Figure 2.**
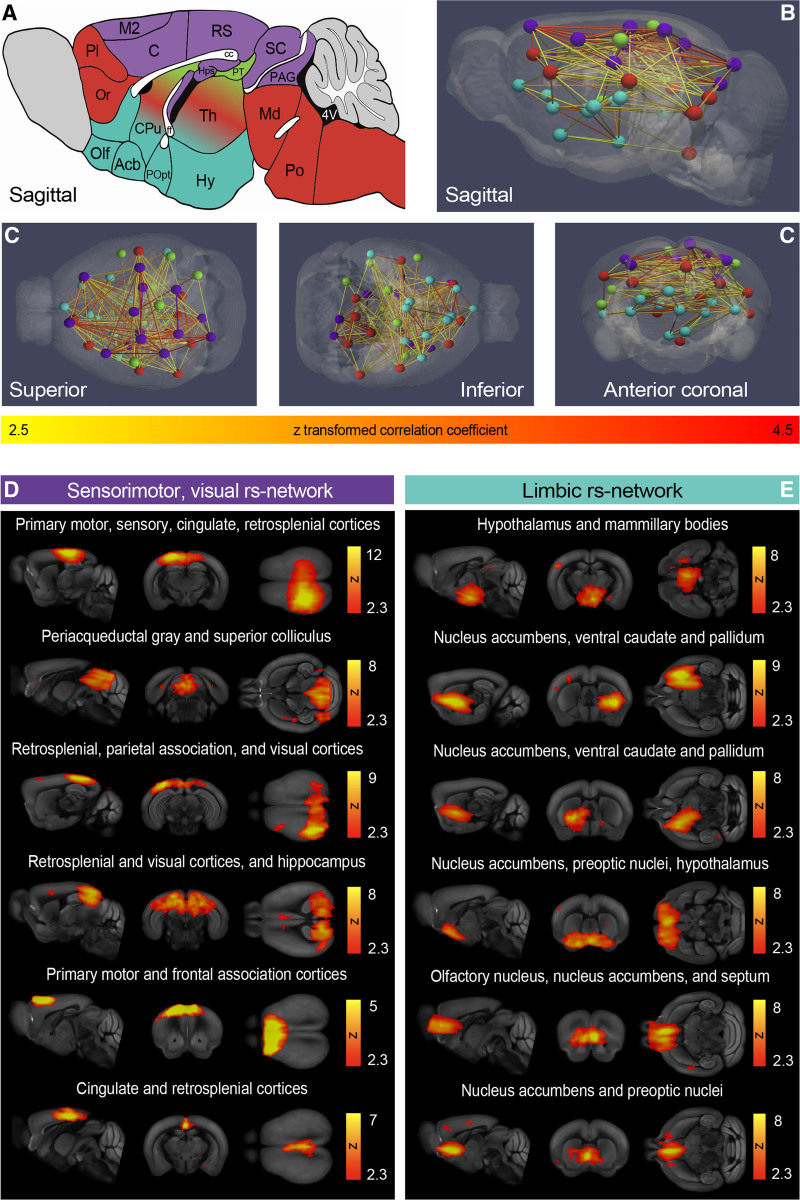
**Mouse-specific resting state networks. A**, Sagittal mouse brain cartoon and (**B**) sagittal, (**C**) superior, inferior, and anterior 3-dimensional views of the 4 color-coded resting state networks: purple: sensorimotor, visual; green: recognition; red: sensory integration and cognition; and blue: limbic. Nodes in **B–E** represent independent activity components, and edges correspond to *Z* scores. **D**, The sensorimotor, visual resting state network contained 12 (6 depicted) and the limbic network (**E**) contained 11 (6 depicted) independent activity components. Independent activity components from the sensorimotor, visual (**D**), and limbic (**E**) resting state networks are overlaid onto the Allen Mouse Brain Atlas; scale bars correspond to the Fisher z transformed correlation coefficient. Acb indicates nucleus accumbens; C, cingulate cortex; cc, corpus callosum; CPu, caudate putamen; ff, fornix; Hy, hypothalamus; Hps, hippocampus; M2, secondary motor cortex; Md, midbrain; Olf, olfactory areas; Or, orbital cortex; PAG, nucleus, periaqueductal gray; Pl, prelimbic cortex; Po, pons; POpt, preoptic nuclei; PT, pretectal nucleus; RS, retrosplenial cortex; SC, superior colliculus; and Th, thalamus.

Independent component analysis identified regions with coherent resting state activity^13^ (n=16). We obtained 59 group independent component analysis components (Figure S3) and 19 were manually classified as noise.^14^ Using a unique approach, hierarchical clustering, the 40 remaining components were grouped according to correlation strength into 4 resting state networks or clusters (color coded in Figure 2). The largest and most highly correlated cluster was primarily cortical. Components spanned the sensory, motor, visual cortices, retrosplenial, and cingulate cortices (Figure 2A through 2G, purple). There were also subcortical components linked to the sensorimotor system (dorsolateral caudate and periaqueductal gray), as well as to visual, spatial function (superior colliculus, hippocampus). This sensorimotor, visual resting state network was anticorrelated with a ventral cluster that consisted of limbic structures: hypothalamus, nucleus accumbens, ventral pallidum, and preoptic nuclei (Figure 2A through 2G, blue). The final 2 resting state networks (recognition and sensory integration and cognition) are depicted in Figure S4 and included components in the sensorimotor cortices, as well as the thalamus, caudate putamen, and brain stem.

### Mice With Vascular Cognitive Impairment Exhibit Decreased Functional Connectivity

**Figure 3.**
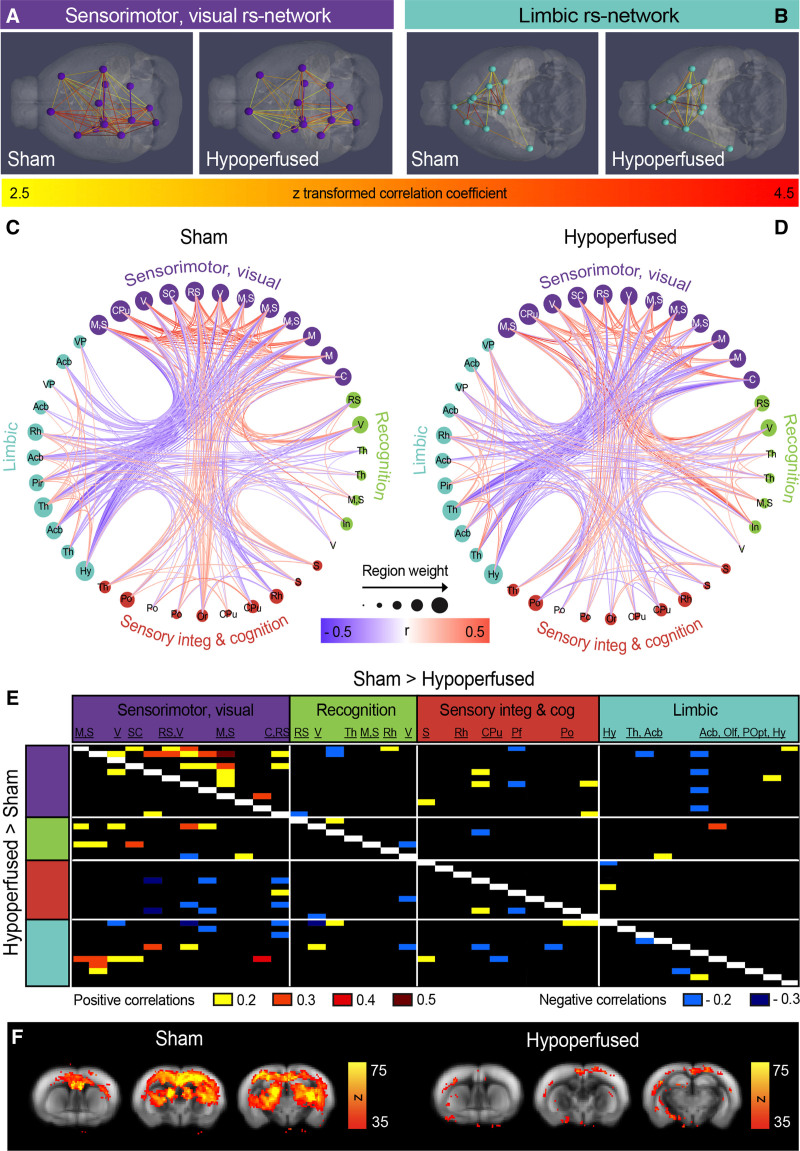
**Functional connectivity changes in hypoperfused mice at 6 mo. A**, Superior, 3-dimensional views of the sensorimotor, visual (**B**), and inferior limbic resting state networks in both groups. Nodes in **A** and **B** represent independent activity components, and edges correspond to *Z* scores. **C** and **D**, Circular views of connectome nodes and edges displaying connectivity in the hierarchical clusters for each group. Nodes represent independent activity components, and node size reflects the number of edges stemming from each node. **E**, Connectivity matrix displaying the strength of the correlation differences between groups (top indicates stronger correlations in shams and bottom stronger correlations in hypoperfused mice). **F**, Cingulate seeding produced a strong cortical rostral-caudal midline activity pattern (rodent default mode network) in shams that was reduced in hypoperfused mice. The default mode network is overlaid onto the Allen Mouse Brain Atlas, and scale bars correspond to the Fisher z transformed correlation coefficient.

There were substantial differences in functional connectivity between the sham and hypoperfused groups in the resting state networks (Figure 3A through 3E). Most notably, correlations among the components in the sensorimotor, visual cluster were weaker in hypoperfused animals compared with shams (Figure 3A and 3B). Circular views of the entire functional connectome highlight this, alongside increases in correlation strengths between the sensorimotor, visual, and recognition resting state networks in hypoperfused mice (Figure 3C and 3D). The negative correlations between the sensorimotor, visual, and limbic resting state networks were also not as strong in hypoperfused mice (Figure 3C and 3D), and these findings were confirmed when differences in correlation strengths between the groups were quantified (Figure 3E). We also observed a rostral-caudal DMN when seed-based analysis from the cingulate cortex was used. When the group data were temporally concatenated, a strong DMN was only observed in the shams (Figure 3F).

### Reorganization of the Functional Connectome Is Accompanied by Progressive White Matter Change in Mice With Vascular Cognitive Impairment

**Figure 4.**
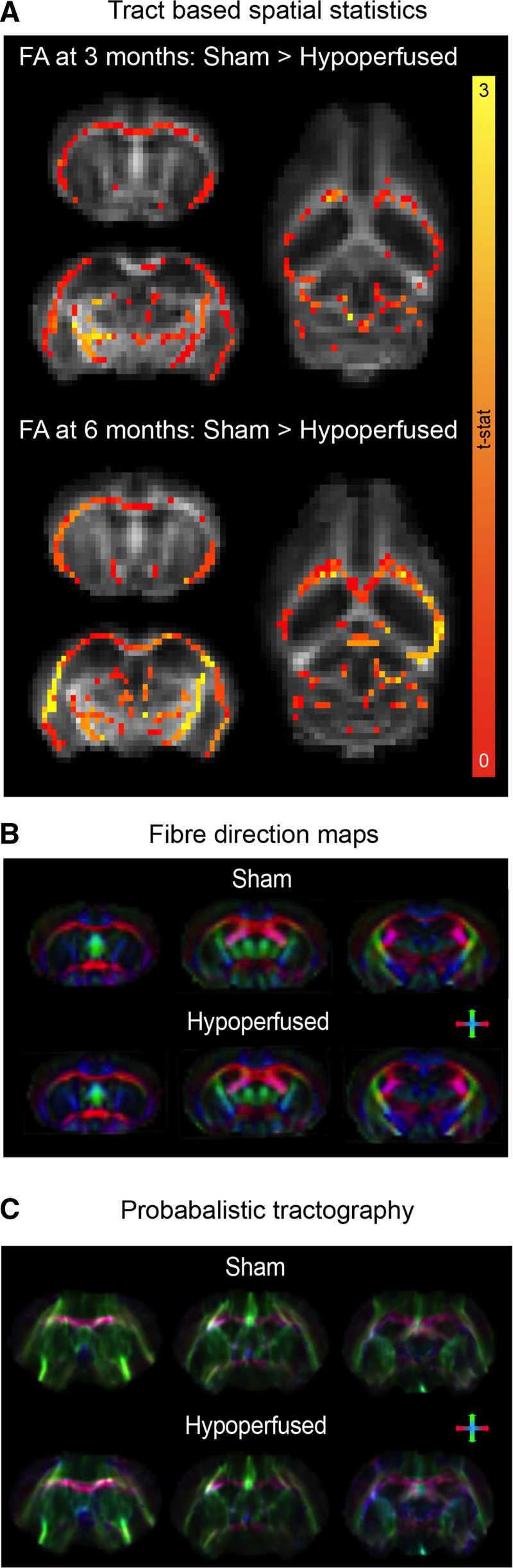
**Progressive white matter change in hypoperfused mice. A**, Tract-based spatial statistics on the mean fractional anisotropy (FA) map revealed shams (n=10) exhibited higher FA values than hypoperfused mice (n=6) in several white matter voxels at 3 and especially 6 m. Scale bar corresponds to t statistic, and entire white matter FA values were sham, 0.665±0.04; hypoperfused, 0.666±0.03 (3 mo); and sham, 0.737±0.03; hypoperfused, 0.714±0.05 (6 mo). **B**, Fiber direction and (**C**) probabilistic tractography images from representative animals. Color depicts direction: pink (left-right), green (superior-inferior), and blue (anterior-posterior).

**Figure 5.**
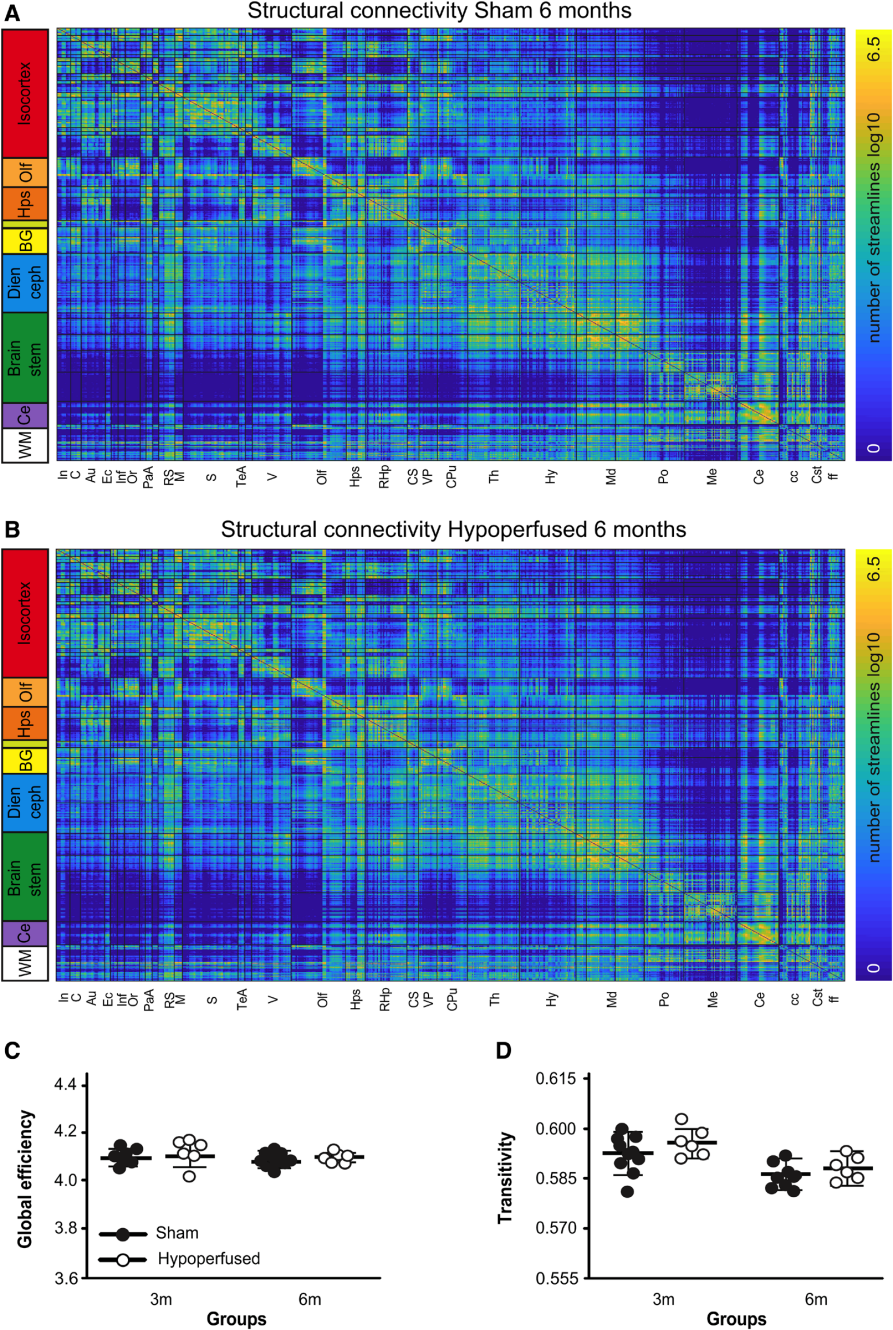
**Structural connectivity is intact in hypoperfused mice.** Group averaged structural connectivity matrices from 681 seeded brain regions are displayed for sham (**A**; n=10) and hypoperfused (**B**; n=6) mice at 6 m. Scale bar corresponds to edge weighting (log of the number of streamlines). **C**, Global efficiency and (**D**) transitivity network properties in sham and hypoperfused groups at 3 and 6 m.

Fractional anisotropy (FA) values throughout the entirety of the white matter were compared between groups using a voxel-wise approach. At 3 months post-surgery, there were clusters of white matter voxels with higher FA values in shams and there were substantively more by 6 months (Figure 4A). This was apparent in the corpus callosum, though none of the voxels remained significantly different after multiple corrections. Fiber direction maps (Figure 4B) and probabilistic tractography images (Figure 4C) were similar between sham and hypoperfused mice. The number of tractography streamlines (not equivalent to fiber density) was used to generate structural connectivity matrices (Figure 5A and 5B). The white matter had a high degree of connectivity with most brain regions. Most cortical regions broadly showed high connectivity, and the somatosensory area had the highest number of streamlines with other cortical regions. The caudate had the highest connectivity. The midbrain was well connected to the motor and retrosplenial cortices, thalamus, and cerebellum. The medulla was poorly connected with most regions except the pons and cerebellum. There were no obvious edge-wise structural connectivity differences between shams and hypoperfused mice at 3 or 6 months (Figure 5A and 5B); this was supported by comparable global efficiencies (Figure 5C). However, there was evidence of regional differences. Interestingly, local efficiency was significantly higher in the visual association cortex of hypoperfused mice (F(1,14)23.996, *P*=0.002), and this survived multiple comparisons (*P*=0.032). Transitivity decreased in both groups at 6 months (main effect of time: F(1,14)20.4, *P*=0.0001).

### Metabolite Changes in Mice With Vascular Cognitive Impairment Suggest Impaired Neuronal Processes

Metabolite concentrations were measured using localized magnetic resonance spectroscopy in the striatum (Table S1). Interestingly, taurine increased in shams and decreased in hypoperfused mice between 3 and 6 months (time×group interaction: F(1,9)6.568, *P*=0.031). Glycerophosphocholine and glutamate+glutamine increased in both groups between 3 and 6 months (main effects of time: F(1,9)8.372, *P*=0.018; F(1,9)20.76, *P*=0.001, respectively), and glutamate+glutamine survived multiple comparisons (*P*=0.012). Lactate and alanine decreased in both groups between 3 and 6 months (main effects of time: F(1,9)7.977, *P*=0.020; F(1,9)12.52, *P*=0.006, respectively), and alanine survived multiple comparisons (*P*=0.036).

## Discussion

We present an image processing pipeline for structural and functional connectivity neuroimaging data in the mouse that is accessible to nonexpert users and uses freely available tools. We identified progressive reductions in FA in the white matter of mice with vascular dementia with little overall change in structural connectivity. However, substantial alterations in functional connectivity were observed. Mice with features of vascular dementia did not exhibit a strong DMN, and connectivity within the sensorimotor, visual resting state network was markedly reduced. The most affected regions participate in visual and spatial memory: the hippocampus and visual cortex. Behavioral data support this finding, as mice exhibited spatial memory impairments even when visual cues were available.

We observed previously reported features of small vessel disease such as chronic microbleeds,^15^ as well as lacunar infarctions that have not been reported in this model but do represent a common radiological feature observed in the clinic. Neither was consistently present, or linked to mortality, suggesting variability in the model severity. The acute infarctions were likely due to the decrease in CBF, but the brain is capable of autoregulation, and CBF recovered to near sham values within 6 months.^16,17^ It is also possible that the increased tortuosity and dilation in the circle of Willis contributed to CBF recovery. Nevertheless, brain and hippocampal atrophy were observed in the hypoperfused group that is indicative of long-term cell loss. While brain atrophy is not previously reported, other groups have observed hippocampal atrophy in response to cerebral hypoperfusion.^17^ The hippocampal degeneration may be partially responsible for the spatial memory deficits in hypoperfused animals.^18,19^ While our data confirm that mice exhibited spatial memory impairments, water maze deficits were present when the escape platform was visible, suggesting additional visual impairments.^20,21^ The functional connectivity data collectively support the behavioral findings as the visual and retrosplenial cortices and hippocampus were the most profoundly affected components between sham and hypoperfused mice.

Hierarchical clustering of functional connectivity data produced 4 highly correlated resting state networks. The most prominent was a sensorimotor, visual cluster that was anticorrelated with a ventral limbic resting state network. A similar hierarchical approach also reported the largest and most highly correlated resting state network in the mouse included sensorimotor, visual, and cingulate cortices, as well as the hippocampus and cerebellum,^22^ and ventral components resulting from independent component analysis have been grouped into striatal and thalamic or limbic resting state networks previously.^8,23^ Even without hierarchical analysis, the strong presence of highly correlated cortical components in mice has been well documented using independent component analysis.^23–25^ A recent multicenter study combined 17 data sets from different laboratories and observed strong cortical representation.^26^ There was also a consistent rostral-caudal DMN when seed-based analysis from the cingulate cortex was used; this has been reported previously.^27^ We observed a similar pattern, though our DMN included some subcortical representation. It is possible our results include additional resting state networks, but we also used a larger seed that encompassed the entire cingulate region.

A key finding of the present study is that the DMN was disrupted in the mice with features of vascular dementia. To the best of our knowledge, no study has reported changes to the mouse DMN in response to cerebrovascular pathology, despite this being a consistent finding in the clinic. Slight variations in functional connectivity strength have been reported in the DMN across the mouse life span,^28^ and doxycycline improved connectivity between the hippocampus and some nodes associated with the DMN in a transgenic mouse model of Alzheimer disease.^12^ In addition to the lack of DMN activity, we also observed functional connectivity differences between the sham and hypoperfused groups in some of the resting state networks. The correlations among the nodes in the sensorimotor, visual cluster were much stronger in shams. This may suggest that prominent cortical networks are more profoundly affected by vascular dementia or that the underlying white matter damage precipitates reductions in intracortical communication. It may also be due to the fact that the most widely affected regions (visual cortex and hippocampus) were well represented in this network. Similarly, a decline in functional connectivity has been detected in the sensorimotor cortices of transgenic Alzheimer disease mice that was apparent as early as 2 months of age.^11^ Despite the functional connectivity differences, overarching small-world network properties remained intact in hypoperfused animals. This suggests that fundamental properties of brain network organization were preserved. While global efficiency (average inverse shortest path length for all pairs of nodes) estimates the brain’s overall ability to integrate information, local efficiency is calculated on the basis of each node. Lower regional local efficiency values suggest that there is little resistance to network failure in those regions, and this has been reported in prefrontal and superior temporal regions in patients with subcortical vascular cognitive impairment.^6,29^ Local efficiency was generally lower across most of the components in the hypoperfused group, though more data are required to determine which regions might be most vulnerable as none survived multiple comparison corrections.

White matter damage has been consistently reported in hypoperfused mice, and our voxel-wise method revealed clusters of reduced FA by 3 months that was much more widespread by 6 months. As none of these voxels survived multiple comparisons, it is possible that the white matter damage may not be as severe as previously reported at more acute time points. While other groups have reported decreases in FA in the white matter around 1 month, the results are inconsistent across structures,^30,31^ and there are also reports of no FA change.^32^ It is possible that the model is not reproducible or that focused region of interest analysis either misses key areas or focuses on them. Our voxel-wise approach enables observation of white matter in its entirety, and although it is subject to multiple corrections, the progressive change supports the idea that damage is occurring slowly. Indeed, more profound decreases in white matter FA in hypoperfused mice have been reported at 6 months.^15^

There are only a few articles that have examined the mouse structural connectome in a disease state. Substantively reduced structural connectivity has been observed in the cuprizone model of demyelination.^33^ This is not surprising given the widespread white matter damage in this model. However, only subtle connectivity changes have been reported in other mouse models of dementia. For example, pairwise interhemispheric connectivity was reduced between the hippocampus and cerebellum of apolipoprotein E APOE4 carrier (a genetic risk factor for Alzheimer disease) compared with APOE3 mice.^34^ As overall structural connectivity appeared more or less unchanged in the hypoperfused mice, it was not surprising that there were no differences between sham and hypoperfused mice in terms of network characteristics. The utility of global efficiency to predict cognitive decline in patients with small vessel disease may be due to the fact that humans have more white matter and are thus more susceptible when damage is concentrated there. However, considering more chronic time points than 6 months in the mouse may be necessary for widespread inefficiencies in the routes for information exchange to develop across the brain network; this is supported by the voxel-wise FA data. It is also possible the rodent brain is capable of compensation to maintain network characteristics. Indeed, no differences in structural or functional global efficiency were observed in 5XFAD (5-familial Alzheimer disease) mice compared with wild types.^35^ The 5XFAD mice exhibited higher path lengths, but only at low network densities, and we chose to maintain network density across both groups with a proportional threshold prior as density is known to influence graph theory parameters. Nevertheless, there is indication from the local efficiency results that connectivity in specific nodes may change in response to hypoperfusion. The increase in local efficiency in the visual association cortex of the hypoperfused group suggests this region is more connected to other brain regions and this may coincide with the decreased functional connectivity observed in the visual cortex.

Metabolite concentrations were measured using localized magnetic resonance spectroscopy in the striatum, and we observed a significant decrease in taurine in the hypoperfused mice at 6 months. In addition to being a profound neuromodulator, taurine stimulates neurogenesis and reduces neuroinflammation and oxidative stress.^36^ It is, therefore, not surprising that taurine reductions were observed in the hypoperfused mice and future work will continue to investigate this possibility. The increased glutamate and glutamine in both groups over time was unexpected, as glutamate tends to decrease with age (attributed to neuronal loss) while glutamine increases.^37,38^ The glutamine increase is presumed to result from the glutamate-glutamine cycle in which glial cells synthesize glutamine from glutamate to maintain homeostasis. It is possible this is still occurring in the present study as, despite the increased field strength, we were unable to resolve separate glutamate and glutamine peaks. The decrease in alanine in both groups with age^38^ may support this as alanine is a nitrogen and ammonia carrier that supports the glutamate-glutamine cycle.^39^ More work would be required to elucidate whether these processes are indeed occurring in the aged brain.

## Conclusions

Overall, we observed additional radiological features of small vessel disease in a mouse model of vascular cognitive impairment that improve the translational potential of the model. This was combined with a trend toward subtle but progressive white matter change without overarching changes in structural connectivity. We are the first to show functional connectivity change in this model. We report little evidence of DMN activity, which matches clinical observations, and a hypothesis-free, data-driven approach revealed decreased functional connectivity in the sensorimotor visual resting state network of hypoperfused mice. It is also possible that the observed decreases in functional connectivity may be compounded by impaired neurovascular coupling or cerebrovascular reactivity, and this should be investigated in future studies. Other limitations of the present study include lack of preregistration and power analysis. Replication of our findings in a larger cohort would be ideal, alongside additional time points to really understand the progression of connectivity change. However, we are encouraged that both the imaging and behavioral data suggest that the visual spatial memory system is vulnerable to vascular insufficiency. This finding also has value, as clinical efforts to use the visual system, specifically the retina, for diagnosis of cerebrovascular disease are underway. The present study advances efforts toward preparation and understanding of the structural and functional mouse connectome and brings novel insight into connectivity alterations in response to vascular disease.

## Article Information

### Acknowledgments

Dr Boehm-Sturm, Dr Dirnagl, M. Foddis, S. Mueller, and Dr Farr conceptualized the research, and Dr Boehm-Sturm, M. Foddis, S. Mueller, Dr Sassi, and Dr Yildirim were involved in data acquisition. Dr Hall, Dr Boehm-Sturm, Dr Finke, M. Foddis, Dr Harms, S.P. Koch, Dr Kuchling, Dr Madan, Dr Sotiropoulos, Dr Trueman, Dr Wallis, and Dr Farr performed the analysis. All authors approved the final manuscript.

### Sources of Funding

This research was funded in whole or in part by the the Biotechnology and Biosciences Research Council (BB/M008770/1), the German Research Foundation (Exc 257, NeuroCURE Cluster of Excellence BO 4484/2-1 to Dr Boehm-Sturm, S.P. Koch, and S. Mueller, and HA5741/5-1 to Dr Harms), the Federal Ministry of Education and Research (01EO1301; Center for Stroke Research Berlin), and the European Commission (01EW1201 and 01EW1811, ERA-NET NEURON).

### Disclosures

Dr Kuchling received congress registration fees from Biogen and speaker honoraria from Sanofi Genzyme and Bayer Schering. Dr Kuchling in the BIH-Charité Junior Clinician Scientist Program was funded by the Charité-Universitätsmedizin Berlin and Berlin Institute of Health. Dr Trueman received a buyout of teaching time from the Wellcome Trust (204843/Z/16/Z) during the preparation of the manuscript.

### Supplemental Material

Supplemental Materials and Methods

Figures S1–S6

## Supplementary Material


