# Supplementary material for: Long-Term Connectome Analysis Reveals Reshaping of Visual, Spatial Networks in a Model With Vascular Dementia Features
Source: Stroke. 2022 Feb 2;53(5):1735–45. doi: 10.1161/STROKEAHA.121.036997 (PMC9022688; doi:10.1161/STROKEAHA.121.036997)
Supplement: Supplementary file 1 [file str-53-1735-s001.pdf]

SUPPLEMENTAL MATERIAL

Supplemental Methods

Behavioural testing

All behavioural tests were recorded and analysed with a computerized automated tracking system (VideoMot 2, TSE Systems, Germany). For novel object recognition, mice were habituated to an empty cage for 10 min on the day prior to testing. During the first trial (10 min) mice were positioned facing away from two identical wooden objects (kept in bedding from unfamiliar mice) secured to the floor. Two hours later, the right object was replaced (stored in bedding from a different group of unfamiliar mice) and mice were allowed to explore for 5 min. Total time spent with each object was calculated, and data was presented as discrimination ratio (right object / (right object + left object)).

The Morris water maze (120 cm diameter circular pool) contained visual cues. A cue task was performed for 4 days with a visible platform in the same location (3 trials of maximum 90 s). If mice failed to escape, they were guided to the platform. Subsequently, a place task was used for 7 days in which the escape platform was submerged (4 trials, 20 min apart, with randomization of entry point). Animals were collected 10 s after locating the platform. A probe trial without a platform was performed on the 7th and 8th day. Escape latency (s), total distance travelled (cm), swim speed (cm/s), and total time spent in each quadrant (s) were measured, and daily data was pooled.

MRI measurements

All MRI measurements were performed using a 7T system (Bruker BioSpin, Germany) under 1.2-1.75 % isoflurane anaesthesia (including for the rsfMRI, but at lower levels 0.75-1.2 %) and temperature and respiration were monitored (SA Instruments, USA). Cerebral blood flow (CBF) was measured with a 72 mm diameter transmit volume coil (RAPID Biomedical, Germany) and a mouse quadrature receive surface coil (Bruker BioSpin) using a 2D Flow-Sensitive Alternating Inversion Recovery (FAIR) sequence (repetition time/echo time (TR/TE): 12 000/35.9 ms, 16 inversion times (Ti): 35-1500 ms, field of view (FOV): 25.6 x 25.6 mm, Matrix: 128 x 64, 1 mm thick single slice, voxel size: 0.08 mm^3, acquisition time: 18:26 min). Time of flight magnetic resonance angiography (MRA) was acquired with a 20 mm diameter quadrature volume coil (RAPID Biomedical, Germany) (TR/TE: 14/2.1 ms, FOV: 20 x 20 x 17.6 mm, Matrix: 150 x 150 x 120, slab size: 0.0026 mm^3, acquisition time: 4:12 min). Spectroscopy, T_2, susceptibility weighted imaging (SWI), rsfMRI, and dMRI were acquired with a transmit/receive cryocoil (Bruker BioSpin, Germany). A STimulated Echo Acquisition Mode (STEAM) sequence was used for 1H MR spectroscopy (TR/TE: 2500/3 ms, VAPOR water suppression, 8 mm^3 striatal voxel, acquisition time: 10:40 min), the T_2 sequence was Rapid Acquisition Relaxation Enhancement (RARE) (TR/TE: 3100/33 ms, FOV: 19.2 x 19.2 mm, Matrix: 192 x 192, 0.5mm slice thickness, voxel size: 0.005 mm^3, acquisition time: 2:48 min), SWI (TR/TE: 700/18 ms, FOV: 19.2 x 19.2 mm, Matrix: 256 x 256, 0.5 mm slice thickness, voxel size: 0.0028 mm^3, acquisition time: 14:44 min), a gradient echo-echo planar imaging (GE-EPI) sequence was used for rsfMRI (TR/TE: 1000/10 ms, 300 repetitions, FOV: 19.2 x 12 mm, Matrix: 128 x 57, 0.75 mm slice thickness, voxel size: 0.02368 mm^3, acquisition time: 8:08 min), and the dMRI data was acquired with an isotropic EPI-DTI sequence (TR/TE: 7500/18 ms, bvals: 0,1000 s/mm^2, 126 directions, gradient duration/separation: 2.5/8.1 ms, FOV: 16.2 x 16.2 mm, Matrix: 72 x 48, 0.225 mm slice thickness, voxel size: 0.017085 mm^3, acquisition time: 67:34 min).

Image analysis

Brain/hippocampal volumes were obtained from T_2 images via manual delineation (ImageJ, NIH, USA), and fractal dimensionality was assessed using the calcFD toolbox (https://github.com/cMadan/calcFD).
CBF maps were calculated via the T_1 method using a blood T_1 of 2100 ms and a brain blood partition coefficient of 0.89 mL/g with the following formula:

$$CBF = \lambda \times \frac{T_{1,nonsel}}{T_{1,blood}} \left(\frac{1}{T_{1,sel}} - \frac{1}{T_{1,nonsel}} \right) * 6000 \ \frac{ml}{min \times 100\ g}$$

All rsfMRI data was converted to NIfTI format using Bru2nii (https://github.com/neurolabusc/Bru2Nii), scaled by a factor of 10, and bias field corrected using N4BiasFieldCorrection (https://github.com/bigbigbean/N4BiasFieldCorrection). All linear within-subject registrations among structural T_2 and functional images were performed using affine transforms in Advanced Normalization Tools (ANTs, version 2.2.0) (https://github.com/ANTsX/ANTs). ANTs was also used for all non-linear registrations to the Allen Mouse Brain Atlas (https://mouse.brain-map.org/static/atlas). Skull stripping was performed with template brain masks warped into individual subject space. The FMRIB Software library (FSL) (www.fmrib.ox.ac.uk/fsl) was used for rsfMRI data analysis. Correction for image drift and motion was performed with MCFLIRT. Data was smoothed by the full width at half maximum (0.7mm kernel) as we were interested in large scale brain networks rather than identifying small regions of activity, and MELODIC was used for independent component analysis (ICA). Neural-driven signal was manually classified at the single subject level as predominantly low frequency (<0.1 Hz), predicted 1.5 – 3 % of the data variance, exhibited no sudden jumps, and activity components needed to be clustered in gray matter (not vasculature, ventricles, or non-brain tissue). Subsequently, 59 components were produced at the group level and 19 were manually eliminated as noise according to the same criteria. Dual regression was performed for 14 components of interest followed by voxelwise testing for group differences using FSL's random permutation testing tool. The p-values in the resulting clusters were Bonferonni corrected (14 comparisons). Masks for seed-based connectivity were generated from the Allen Mouse Brain Atlas.

The MATLAB (version R2019a) toolbox FSLNets (version 0.6) (www.fmrib.ox.ac.uk/fsl) was used to generate all rsfMRI weighted connectivity matrices. For visualization of connectivity matrices, glass brains were prepared in ParaView (version 5.6.0) (https://www.paraview.org/) from 3D renderings of the surface of the mouse brain prepared from a whole brain mask in ITK-SNAP (version 3.6.0) (http://www.itksnap.org/pmwiki/pmwiki.php). Individual components were manually thresholded and exported as binarized masks into ParaView. The masks were converted into spheres, and Fisher's z transformed correlation coefficient edges were added and color coded manually. A proportional threshold was applied across the connectivity matrices that ensured the highest small world value (highest clustering and shortest path lengths) was preserved while maintaining matrix density. The MATLAB (version R2018a) Brain Connectivity Toolbox (https://www.nitrc.org/projects/bct) was used for graph theory and global efficiency, modularity and transitivity were calculated for each animal as well as nodal local efficiency. Circlize visualization in R (https://github.com/jokergoo/circlize) was used to generate the circular views of connectome nodes and edges based on correlation strength (r).

All dMRI data was converted to NIfTI format with Bruker2nifti (https://github.com/SebastianoF/bruker2nifti) followed by bias field correction as per the rsfMRI data. B0 template creation and linear affine registration of individual subject data to the B0 template was performed with ANTs, as were all non-linear transforms to the Allen Mouse Brain Atlas. Template brain masks were used for skull stripping via warping into individual subject space. Image pre-processing, correction, and analysis were performed with FMRIB's Diffusion Toolbox (FDT) (https://fsl.fmrib.ox.ac.uk/fsl/fslwiki/FDT). Scalar maps were generated using DTIFIT. Tract Based Spatial Statistics (TBSS) was used to produce a mean fractional anisotropy (FA) map and a white matter skeleton (threshold of 0.4) for all mice. This was used for voxelwise comparisons of tissue microstructure between groups with Randomise. The Threshold Free Cluster Enhancement option was used to define clusters and p-values were corrected for multiple comparisons using family wise error. For probabilistic tractography, Bedpostx was used to fit a fibre orientation model to each voxel. Probtrackx2 was used with the probabilistic tractography network option. Brain regions were used as seeds and 5000 seeds were placed in each voxel. The step length was half of the voxel size, the angular threshold was 50 degrees, and streamlines were counted if they passed through another atlas region. The resulting structural connectivity matrices reflected the number of streamlines connecting all pairs of regions, and a $\log_{10}$ transform was used to normalize for the distance bias in tractography. Graph theory was performed as per the rsfMRI data, including data appropriate proportional thresholding.

Spectroscopic metabolite concentrations were calculated using water scaling to signal in a water-unsuppressed reference scan in LCModel and values with a standard deviation < 25 % were excluded (1 sham and 2 hypoperfused mice at 3 months).

Supplemental Results

Mice with vascular cognitive impairment display radiological features of small vessel disease

Atrophy was observed in the hippocampus of hypoperfused mice (main effect of group: $F(1,13)5.4$, $p=0.037$, time: $F(2,26)1.7$, $p=0.208$, and time x group interaction: $F(2,26)1.1$, $p=0.4$) (Supplementary Figure IB). Fractal dimensionality is a measure of structural complexity that is more sensitive to inter-individual differences than volume measurements. There were no significant changes in fractal dimensionality between groups over time in the brain (main effect of group: $F(1,14)0.6$, $p=0.458$, time: $F(1,14)0.6$, $p=0.457$, Greenhouse-Geisser, and time x group interaction: $F(1,14)0.584$, $p=0.458$) or hippocampus (main effect of group: $F(1,14)0.7$, $p=0.404$, time: $F(1,14)0.6$, $p=0.451$, Greenhouse-Geisser, time x group interaction: $F(1,14)0.5$, $p=0.486$) (Supplementary Figure IC-D). The lack of differences in structural complexity of the brain or the hippocampus indicates that mice do not have sufficient shape detail for fractal dimensionality.

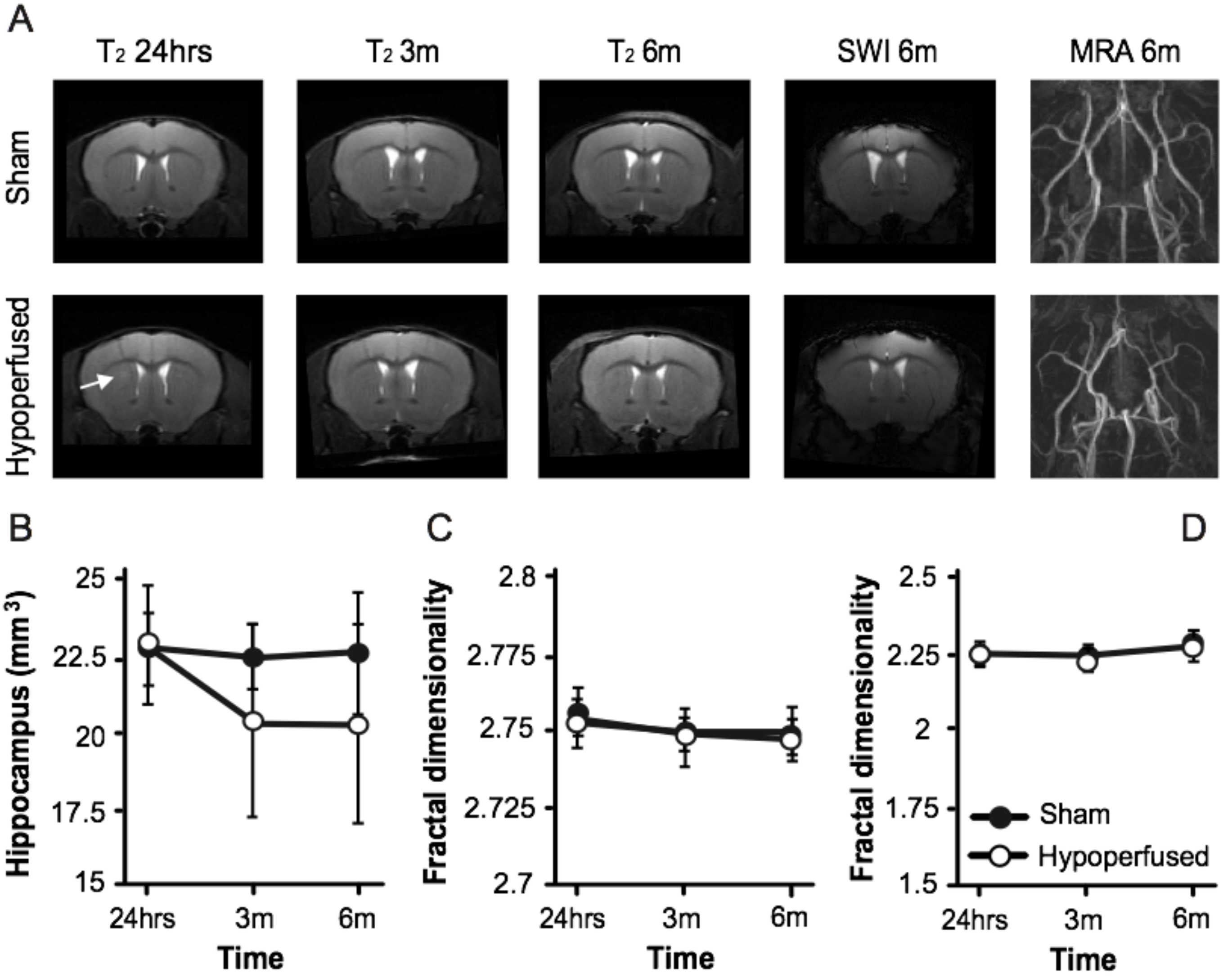

Figure I. Hypoperfusion caused lacunar infarctions, arterial remodeling and brain and hippocampal atrophy without affecting fractal dimensionality. (A) Representative T_2 images from a sham and hypoperfused animal (arrow indicates a lacunar infarction) throughout the course of the experiment alongside a susceptibility weighted image (SWI) and a magnetic resonance angiography (MRA) image of the Circle of Willis at 6m. (B) Hippocampal volume and fractal dimensionality in the brain (C) and hippocampus (D) of sham (n=10) and hypoperfused (n=6) mice throughout the course of the experiments.

Behavioral deficits in mice with vascular cognitive impairment suggest visual, spatial disturbance

In a water maze place task (hidden, fixed platform), escape latencies (F(6,84)2.7, p=0.019), total distance travelled (F(6,84)3.9, p=0.002), and swim speed (F(2.9,40.2)2.3, p=0.0001) decreased with time (Supplementary Figure IIA-C). Hypoperfused mice did not escape as quickly (F(1,14)42.8, p=0.0001, no interaction), and they travelled greater distances to find the platform (F(1,14)25.6, p=0.0001, no interaction), and swam slower (F(1,14)15.6, p=0.001, no interaction) (Supplementary Figure IIA-C). During the probe trial (removed platform), there were no significant group differences for total time spent in the target quadrant (t(14)1.5, p=0.15; normally distributed D(16)0.151, p=0.200 and equal variance F(14)0.130, p=0.724) (Supplementary Figure IID). However, shams swam further (t(14)3.2, p=0.006; normally distributed D(16)0.122, p=0.200 and equal variance F(14)0.001, p=0.978), faster (t(14)3.2, p=0.006; normally distributed D(16)0.121, p=0.200 and equal variance F(14)0.001, p=0.978), and made more returns to the platform location (t(14)3.6, p=0.003; normally distributed D(16)0.232, p=0.021 and equal variance F(14)0.007, p=0.936) (Supplementary FigIIE). During novel object recognition, all mice explored both objects equally during the first trial, and spent more time with the novel object during the second trial (main effect of time: F(1,14)133.4, p=0.0001). This was not influenced by hypoperfusion (no main effect of group or interaction) (Supplementary Figure 2F).

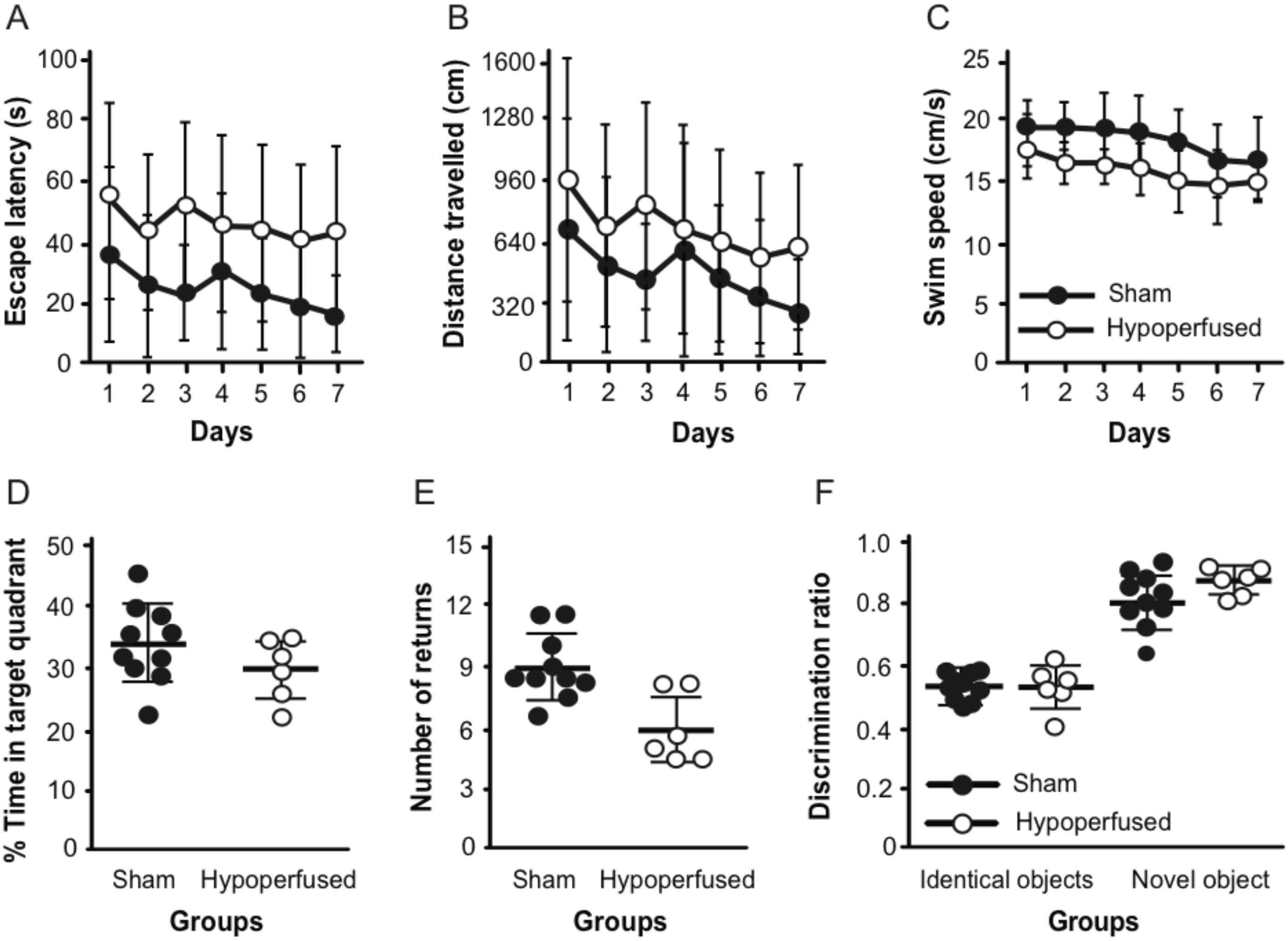

Figure II. Hypoperfused mice exhibit deficits in the water maze without impairments in recognition memory. Escape latency (A), total distance travelled (B) and swim speed (C) in a place task in the Morris water maze for hypoperfused (n=6) and sham (n=10) groups. Time spent in (D) and number of returns to the target quadrant (E) during the probe trial with no platform. (F) Discrimination ratio of both groups in the NOR task.

Characterization of the functional mouse connectome depicts resting state networks

There is uncertainty in the rodent literature surrounding the appropriate number of components for ICA. Specification of low numbers of components (15 and 30) resulted in large areas of activation with limited regional specificity (Supplementary Figure III). Ultimately, a data driven, automated approach was employed in which principle component analysis chose the number of components that reflected a predictable amount of variance that can be explained according to data quality.

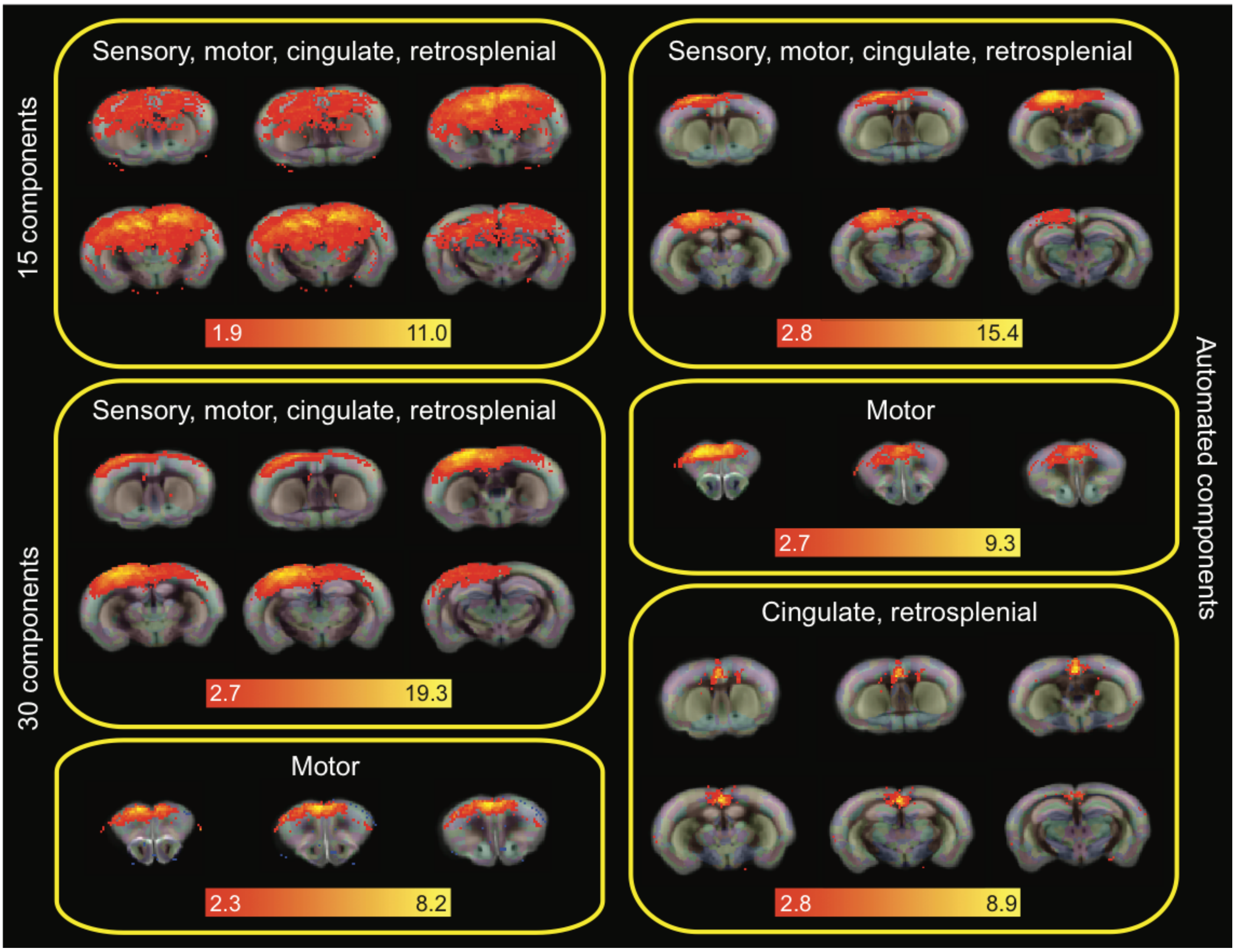

Figure III. Variation in the number of selected components. The same sub-network (sensory, motor, cingulate, and retrosplenial cortices) is depicted under an analysis that specified 15 (top left), 30 (middle and bottom left), and an automatically determined number of components (right). A motor cluster is depicted from the 30 and automated analysis (bottom left and middle right, respectively) alongside additional components in the cingulate and retrosplenial cortices that were detected from the automated analysis. Note: Components are overlaid onto the Allen Mouse Brain Atlas and scale bars correspond to the Fisher's z transformed correlation coefficient.

The recognition, and sensory integration and cognition resting state networks are depicted in Supplementary Figure IV.

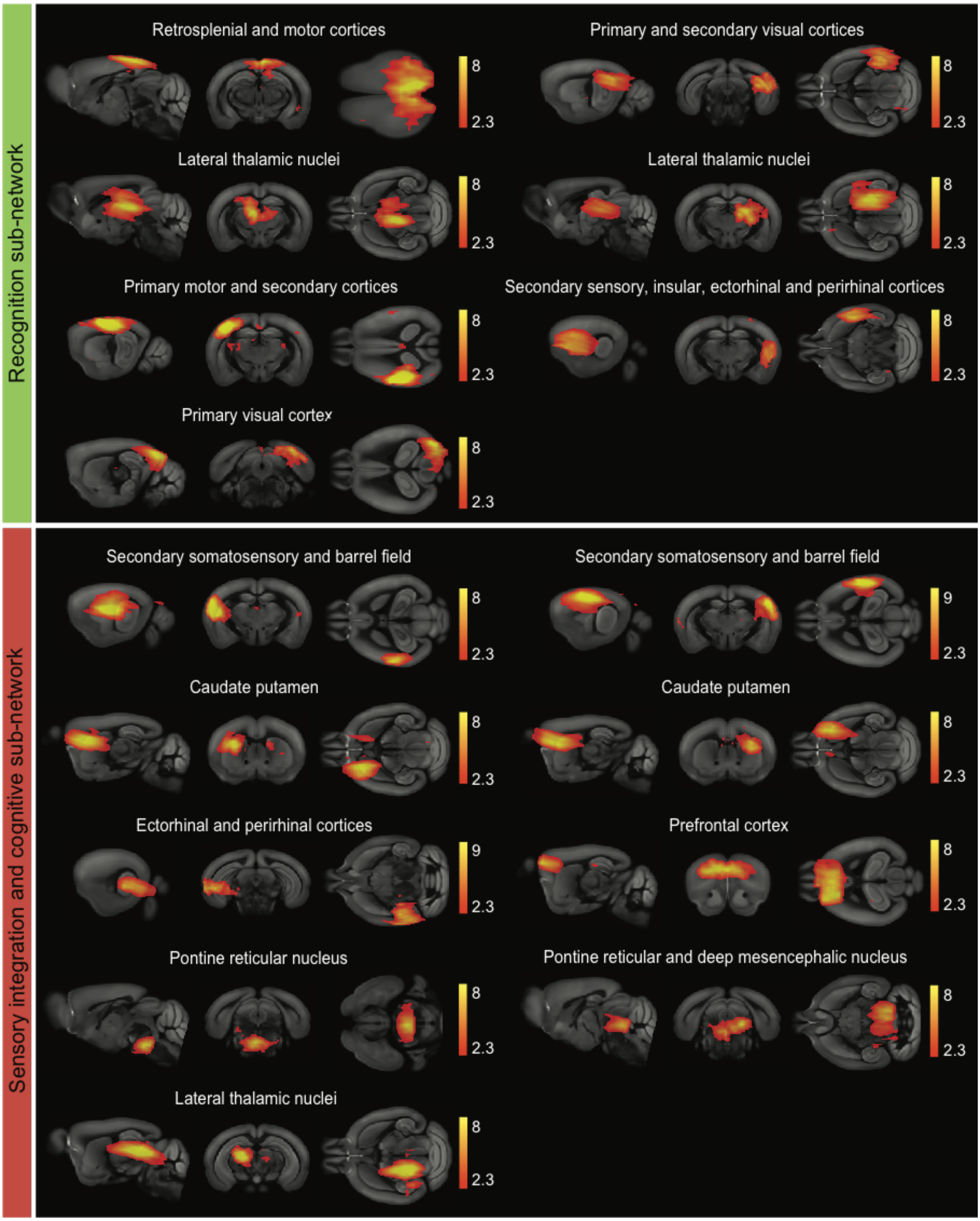

Figure IV. The recognition and sensory integration and cognition resting state networks. The recognition (green) contains 7 and the sensory integration and cognition (red) sub-network contains 9 independent activity components that are highly correlated. Note: Components are overlaid onto the Allen Mouse Brain Atlas and scale bars correspond to the Fisher's z transformed correlation coefficient.

The recognition resting state network included cortical (retrosplenial, visual, and sensorimotor) and lateral thalamic components (Supplementary Figure IV, green). The retrosplenial cortex has reciprocal connections with the sensory, motor and visual cortices, as well as the anterolateral thalamus. These structures play a role in integration of information, particularly in the context of spatial navigation (learning locations, recognizing landmarks). There are also reciprocal connections between the visual cortices and lateral thalamic nuclei.

The sensory association and cognition resting state network (Supplementary Figure IV, red) contains activity components in the secondary somatosensory and prefrontal cortices (including the anterior cingulate), as well as the caudate and aspects of the midbrain and pons. The secondary somatosensory cortex is reciprocally connected to the prefrontal cortex. The former is a major integrative structure when it comes to processing sensory information, while the latter is involved with performance adjustment and modulating behaviors. There are also projections to the medial caudate from the prefrontal cortex that are associated with sensory and cognitive processes. The functions of the pontine tegmental area and deep mesencephalic nucleus are not well characterized, but in addition to being associated with sleep and consciousness, they receive striatal input and may participate in perception, vigilance and awareness associated with cognitive basal ganglia behaviors.

Mice with vascular cognitive impairment exhibit decreased functional connectivity

A hypothesis driven approach selected 14 components of interest for follow up: the hippocampus and visual cortex, along with components that corresponded to regions that are highly connected to both structures: thalamus, hypothalamus, and retrosplenial cortex. We also included components in the caudate and orbital frontal cortex as we were interested in frontal striatal hubs associated with cognition in the mouse. Dual regression of the group-ICA components and voxelwise testing of the spatial maps from the 14 components of interest was used to probe for functional connectivity differences between groups. The hypothesis was rejected in clusters associated with 3 of the selected components: the hippocampus, one of the visual cortex components, and the retrosplenial cortex (Supplementary Figure VA). The hippocampal and visual cortex components explained 2.34 % and 2.17 % of data variance, respectively. This suggests differences in functional connectivity in these components between sham and hypoperfused mice, despite a lack of voxelwise significance after 14 multiple comparison corrections.

When overall network characteristics were estimated, there were no significant differences between groups for global efficiency ($U=26$, $p=0.713$; not normally distributed $D(16)0.250$, $p=0.009$ but equal variance $F(14)0.213$, $p=0.652$), modularity ($t(14)0.187$, $p=0.854$; normally distributed $D(16)0.111$, $p=0.200$ and equal variance $F(14)1.205$, $p=0.291$), or transitivity ($U=16$, $p=0.147$; not normally distributed $D(16)0.291$, $p=0.001$ but equal variance $F(14)0.012$, $p=0.915$) (Supplementary Figure VB-D). Correlations between global efficiency and water maze performance (area under the curve for the cued task) were low ($r=0.19$) and moderate for characteristic path length $r=0.30$.

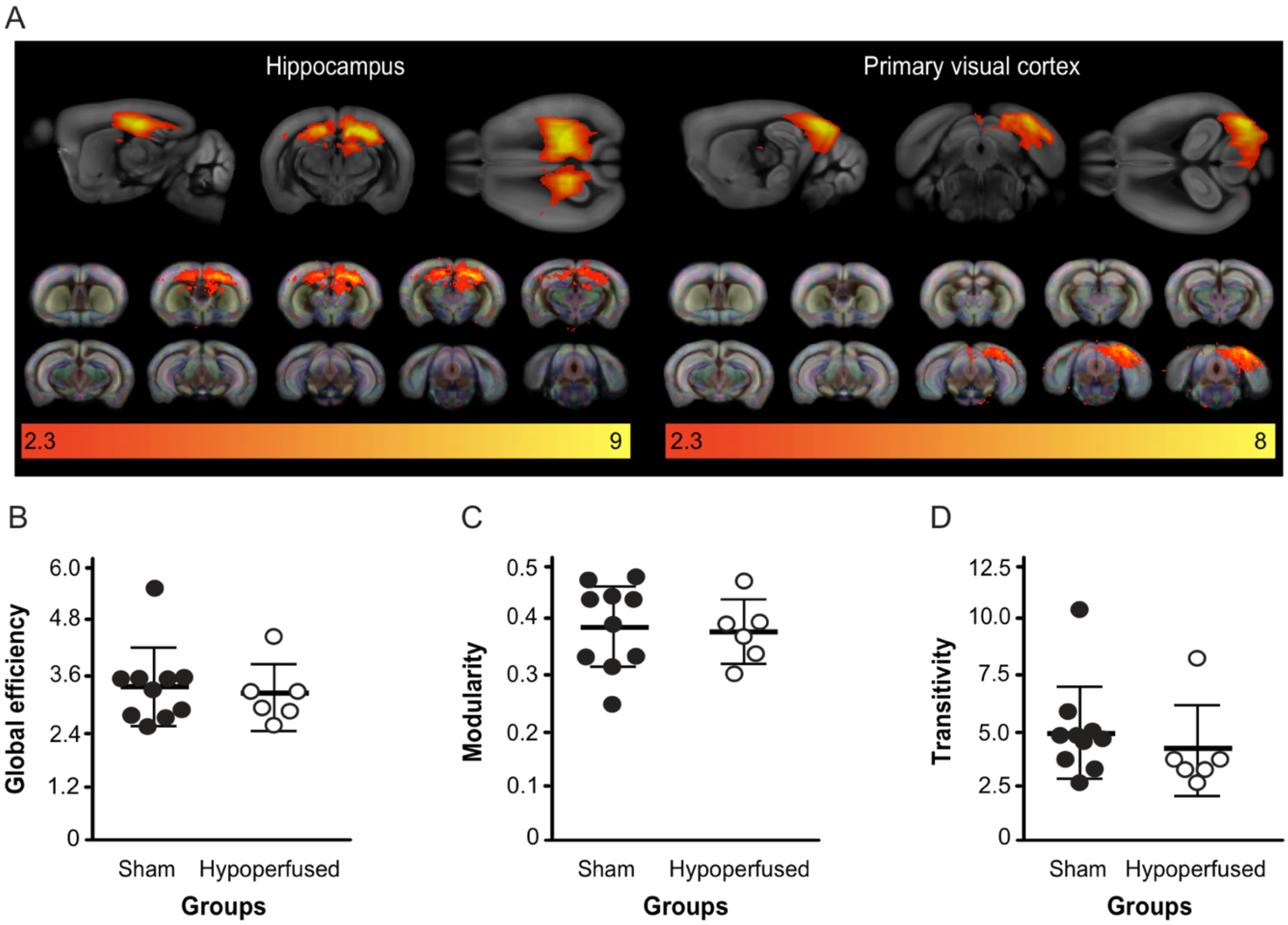

Figure V. The hippocampus and visual cortex were the most affected brain regions though the entire network exhibited preserved characteristics. (A) Hippocampus and visual cortex components exhibited connectivity differences between the sham and hypoperfused groups. Note: Components are overlaid onto the Allen Mouse Brain Atlas and scale bars correspond to the Fisher's z transformed correlation coefficient. (B) Global efficiency, (C) modularity and (D) transitivity network properties in sham (n=10) and hypoperfused (n=6) groups.

Re-organization of the functional connectome is accompanied by progressive white matter change in mice with vascular cognitive impairment

The 681/1206 Allen Mouse Brain Atlas regions that survived down-sampling for probabilistic tractography are still well below the resolution of the dMRI and. Up to 90% similarity between dMRI structural connectomes and the Allen Mouse Brain Atlas has been reported when a courser atlas parcellation of 96 regions was used. Therefore, we combined the Allen Mouse Brain Atlas regions into only the parent structures (32 regions). Despite this, there were still no obvious edge-wise structural connectivity differences between groups at either 3 or 6 months (Supplementary Figure VI).

Graph theory was used to examine overall structural network characteristics. There were no significant differences in modularity between groups at either 3 or 6 months (main effect of group: $F(1,14)2.6$, $p=0.127$, time: $F(1,14)2.9$, $p=0.109$, time x group interaction: $F(1,14)0.02$, $p=0.890$). However, several nodes exhibited reduced local efficiency in both groups at 6 months (main effects of time). The corpus callosum ($F(1,14)45.284$, $p=0.0001$), striatum ($F(1,14)28.351$, $p=0.0001$), prelimbic area ($F(1,14)24.144$, $p=0.0001$), hippocampus ($F(1,14)12.861$, $p=0.0003$), corticospinal tract ($F(1,14)6.816$, $p=0.021$), olfactory regions ($F(1,14)19.337$, $p=0.001$), visual cortex ($F(1,14)14.077$, $p=0.002$), pons ($F(1,14)13.368$, $p=0.003$), hypothalamus ($F(1,14)11.329$, $p=0.005$) and visual association cortices ($F(1,14)7.78$, $p=0.014$) All survived false discovery rate multiple comparison corrections ($p=0.001$, $p=0.001$, $p=0.001$, $p=0.002$, $p=0.005$, $p=0.005$, $p=0.009$, $p=0.012$, $p=0.01$, and $p=0.04$, respectively).

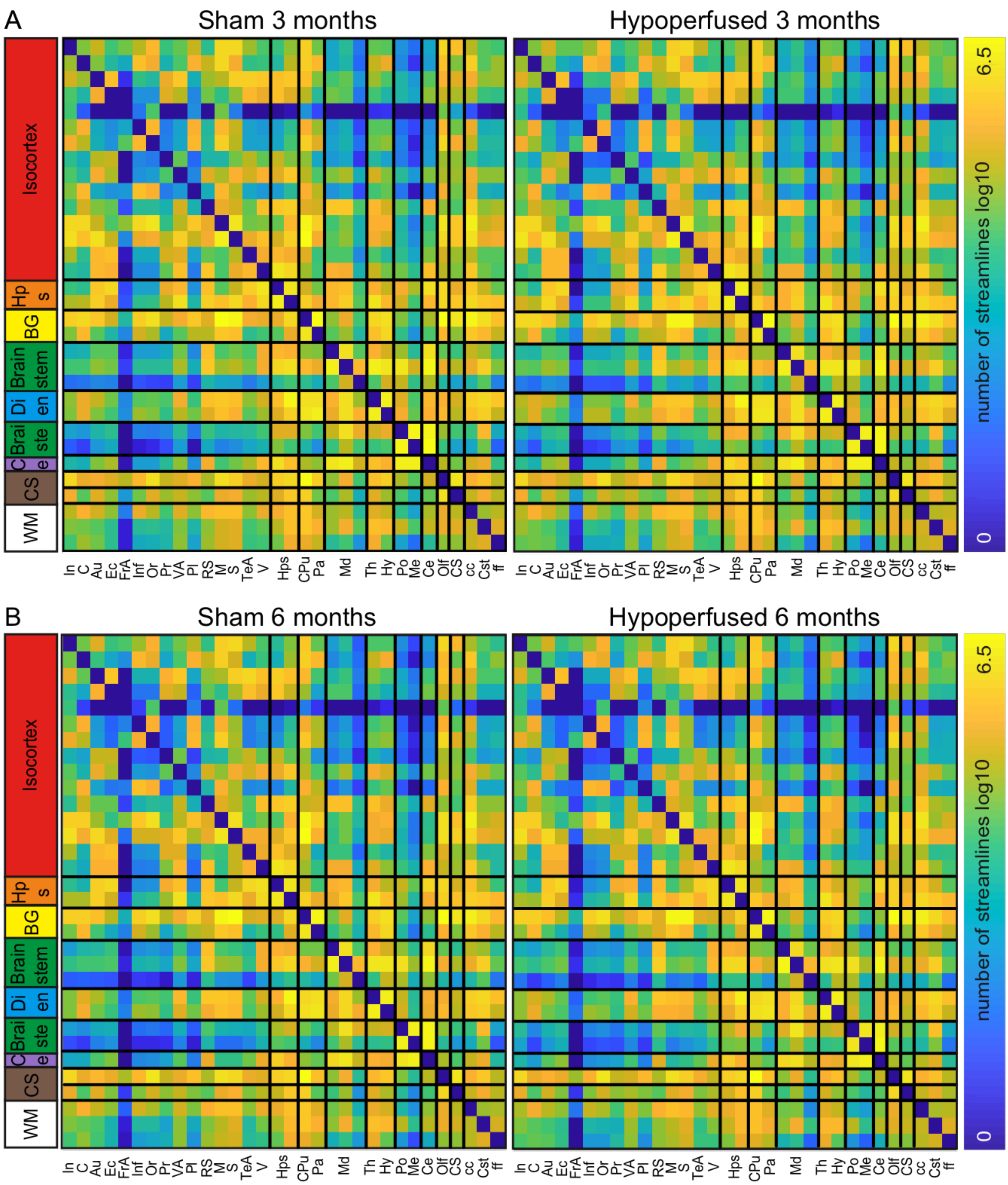

Figure VI. Structural connectivity is intact in hypoperfused mice at both timepoints. Structural connectivity matrices from 32 seeded parent regions are displayed for sham and hypoperfused mice at 3 and 6m. Note: scale bar corresponds to edge weighting (the log of the number of streamlines between each region). Summary regions (left) correspond to higher level collective groups from the Allen Mouse Brain Atlas (http://mouse.brain-map.org/static/atlas) (Isocortex, Hippocampal formation, Cerebral nuclei (basal ganglia), Midbrain, Hindbrain (diencephalon and brain stem), Cerebellum, Olfactory areas, Cortical subplate, and Fibre tracts (white matter). Abbreviations: olfactory areas (Olf), hippocampus (Hps), basal ganglia (BG), diencephalon (Dienceph), cerebellum (Ce), white matter (WM), insular (In), cingulate (C), auditory (Au), ectorhinal (Ec), infralimbic (Inf), orbital (Or), parietal association (PaA), retrosplenial (RS), motor (M), sensory (S), temporal association (TeA), and visual cortices (V), olfactory (Olf) areas, retrohippocampal (RHp) region, cortical subplate (CS), ventral pallidum (VP), caudate putamen (CPu), thalamus (Th), hypothalamus (Hy), midbrain (Md), pons (Po), medulla (Me), cerebellum (Ce), corpus callosum (cc), corticospinal tract (Cst), fornix (ff).

Metabolite changes in mice with vascular cognitive impairment suggest impaired neuronal processes

Metabolite concentrations were measured using localized MR spectroscopy in the striatum (Supplementary Table I).

Table I. Brain metabolite concentrations (mM) in sham and hypoperfused mice across 6 months.

	Sham n(%)			Hypoperfused n(%)			
	3months 7(70)	6months 9(90)	% change	3months 4(36)	6months 6(55)	% change	p value
N-acetylaspartate (NAA)	2.7 ± 0.09	2.78 ± 0.14	4.3	2.74 ± 0.28	2.72 ± 0.09	-0.8	
Creatine (Cr)	1.76 ± 0.09	1.69 ± 0.15	-4.2	1.58 ± 0.24	1.70 ± 0.11	7.5	
Phosphocreatine (PCr)	1.15 ± 0.15	1.36 ± 0.14	16.3	1.29 ± 0.32	1.33 ± 0.13	2.7	
Glycerophosphocholine (GPC)	0.22 ± 0.04	0.31 ± 0.07	1.7	0.24 ± 0.07	0.31 ± 0.05	26.4	$F(1,9)8.372$, $p=0.018$ *
Phosphocholine (PCh)	0.48 ± 0.05	0.44 ± 0.07	-7.4	0.47 ± 0.12	0.41 ± 0.09	-12.2	
Glutamate (Glu) and Glutamine (Gln)	4.11 ± 0.17	4.83 ± 0.26	16.1	4.20 ± 0.35	4.75 ± 0.19	12.3	$F(1,9)20.76$, $p=0.001$*
Inositol (Ins)	1.99 ± 0.18	1.91 ± 0.19	-4.0	2.15 ± 0.32	1.99 ± 0.11	-7.5	
Glucose (Glc)	0.68 ± 0.32	0.82 ± 0.19	17.8	0.60 ± 0.26	0.76 ± 0.12	23.6	
N-Acetylaspartylglutamate (NAAG)	0.02 ± 0.02	0.04 ± 0.04	61.3	0.02 ± 0.03	0.02 ± 0.03	-14.4	
Taurine (Tau)	4.44 ± 0.16	4.74 ± 0.33	6.4	4.55 ± 0.28	4.43 ± 0.16	-2.6	$F(1,9)6.568$, $p=0.031$ ‡
Alanine (Ala)	0.76 ± 0.12	0.56 ± 0.10	-30.7	0.74 ± 0.17	0.54 ± 0.06	-32.1	$F(1,9)12.52$, $p=0.006$ *
Lactate (Lac)	2.38 ± 0.49	1.86 ± 0.28	-24.4	2.12 ± 0.39	1.66 ± 0.16	-24.1	$F(1,9)7.977$, $p=0.020$ *

* Indicates significant effect of time, ‡ indicates significant time x group interaction
